# Reimagining Rural: Shifting Paradigms About Health and Well-Being in the Rural United States

**DOI:** 10.1146/annurev-publhealth-052020-123413

**Published:** 2021-12-15

**Authors:** R.A. Afifi, E.A. Parker, G. Dino, D.M. Hall, B. Ulin

**Affiliations:** 1 Department of Community and Behavioral Health, and Prevention Research Center for Rural Health, College of Public Health, University of Iowa, Iowa City, Iowa, United States; 2 Department of Social and Behavioral Sciences, and West Virginia Prevention Research Center, School of Public Health, West Virginia University, Morgantown, West Virginia, United States; 3 Office of the Associate Director for Policy and Strategy, Centers for Disease Control and Prevention, Atlanta, Georgia, United States; 4 Division of Population Health, National Center for Chronic Disease Prevention and Health Promotion, Centers for Disease Control and Prevention, Atlanta, Georgia, United States

**Keywords:** rural, participation, strength-based, health equity

## Abstract

Rural health disparities have attracted increased national attention, compelling an expanded focus on rural health research. In this article, we deconstruct the definitions and narratives of “rural” communities and suggest that a paradigm shift is needed that centers the complexity and strength of rural places. We discuss the relevance of health equity frameworks, implementation science, and community-engaged approaches to promote rural well-being. Focusing on rural in its own right will lead to intervention innovations and reinvention with implications beyond rural areas. We conclude with suggestions for research and practice to inspire renewed interest in partnering with rural communities to promote health equity.

## THE POLITICS OF PLACE: INTERROGATING “RURAL”

Place matters. Place can create equity or inequity. As Cummings and colleagues note, the representation of place is not neutral, but rather the result of complex political and social processes and choices, centered in power and privilege (27). Narratives of “rural” places represent this complexity as they have been constructed from a metro-normative urban-centric perspective (77), despite the fact that 97% of the land in the United States is rural and 19% of the population live in rural areas (123). Dominant representations of rural places suggest a singular deficit-based place, perhaps captured most vividly in the “deaths of despair” narrative that is used to describe a reversal of mortality declines for White Americans aged 25–54 years who live in rural areas (16, 30). Yet, rural places are nuanced, thriving spaces “where people are born, live, learn, work, play, worship, and age” (49). Understanding the complexity of rural is critical to the development of more responsive social and health policy and services that promote health equity (85).

In this article, we review the definitions, nuance, and diversity of rural areas; examine rural health inequities; and acknowledge the structural determinants that lead to these inequities. We then reimagine the concept of rural with an intent to shift the current paradigm about what constitutes rural and to promote renewed interest in partnering with rural communities to promote health and well-being through research, practice, and policy.

## DEFINITIONS: WHAT IS RURAL?

### US Government Definitions

The US government uses multiple definitions of rural, and three of the most frequently cited are described here. The Census Bureau defines rural as “encompass(ing) all population, housing, and territory not included within an urban area” (124). Urban areas are composed of two types: urban areas that include populations of 50,000 or more, and urban clusters that include populations between 2,500 and 49,999 (124). On the basis of this definition, approximately 20% of the US population in 2010 lived in rural areas. Several changes to the definition were proposed for 2020, including increasing the lower bound of the population to 10,000 and adopting a housing unit density measure rather than a raw population count (125).

The Office of Management and Budget (OMB) designates counties as metropolitan (population size 50,000 or more), micropolitan (population size 10,000–<50,000), and neither. In this classification, any place not classified as metropolitan is considered rural (including those that are micropolitan); about 15% of the 2010 US population lived in rural areas as per this definition (54). Finally, the Federal Office of Rural Health Policy uses rural–urban commuting areas (RUCAs)—which are calculated using population density, urbanization, and commuting—to define rural areas. Places are classified as metropolitan, micropolitan, small town, or rural along a 1–10 scale (126), with about 18% of the population living in rural areas (54). The one commonality across all definitions is that rural is what is left over after urban is defined.

### Other Definitions

Publications in the academic and practice literature use the above definitions and/or create their own definitions. Many of these other definitions of rural also depend on their relative comparison to urban, suggesting that rural is valid only from an urban viewpoint (89, chapter 5). For example, urban areas are often defined precisely, whereas rural areas are all those not urban or with less population or at some distance from urban areas (10). At the same time, people’s conception of urban is diverse, whereas their visual image of rural is singular, often white men and farmers (10). Some definitions are also developed from an economic perspective (e.g., commuting time for employment) and thus are insufficient for a broader understanding of well-being (33).

Definitions of rural also rarely explore the subjective lived descriptions or conceptualizations of rural life that residents possess. Recent scholarship recommends crafting definitions on the basis of a “ground-truthing” mixed methods approach that considers “localities, representations, and everyday lives” (89, chapters 8, 9) and allows the capturing of how context influences health [e.g., “gets into the body” (27)]. Furthermore, rural health scholars emphasize understanding the influences of the context of place on well-being and how that might change over time, by subpopulation, by stage of life (what is salient for youth may be different from what is salient for adults), and by vertical influences (from local to global) (27).

Comparisons between rural and urban areas often presume that all rural places and spaces are the same and create “geographic imaginaries,” which stereotype people, environment, culture, and practices by others who do not occupy rural spaces (64). This dichotomization of the rural–urban divide has dominated conceptualizations of urban and rural spaces. Yet, the continuity of rural–urban spaces is likely a more relevant way to understand the complexity of rural life and the impact of geography, culture, and tradition on health. As Meserole (82) noted in his discussion of rural, “It is not possible to justly indicate the place at which rurality ends—or where urban conditions begin” (p. 233).

## THE NUANCE OF RURAL

These dichotomized definitions flatten the complexity of rural living, minimize the strengths and assets in rural areas, and disregard the diversity and “breadth of demographic, social, economic, and health system characteristics…of rurality” (10, p. 1987; 86; 91; 105). But rural areas across the United States are not (all) created equal, and residents within rural areas have different social and health-related profiles (48, 60). Though most rural areas have experienced economic hardship for many years, and have not been the focus of improvement efforts (71), they are not monolithic. Rural areas across the country have different racial/ethnic profiles, which historically include Native and Black populations along with White persons. More recently, rural areas have increasingly become more racially and ethnically diverse, with the largest growth among Latinx populations (85). Rural areas also range from deeply impoverished to some of the richest counties in the United States (85). And contrary to dominant narratives, farming and mining counties account for only 25% of all rural counties (100). Furthermore, even within these two industries, dominant narratives are inaccurate: Farm industries and workers have taken over family farms (110), and mountain-top mining has taken over underground mining, benefiting coal mining companies and increasing unemployment (51).

Ulrich-Schad & Duncan (122) expand the nuance of rural by suggesting three distinct categorizations of rural areas: amenity-rich, transitioning, and chronically poor. Amenity-rich areas are experiencing population growth owing to (mostly) college-educated newcomers arriving for recreation or natural beauty. These areas are not experiencing the economic decline that is presumed for all rural areas. However, they are not without challenges: Cost of living is increasing in these areas, and identity concerns are emerging between longtime residents and newcomers. Transitioning areas depend on agriculture, timber, and manufacturing. As their name suggests, these areas are transitioning to either amenity-rich or chronically poor but currently are experiencing population decline, particularly among young workers. These are places that have been negatively affected by expanding urbanization and economic restructuring and can be described as the “heart of the rural US” (122, p. 62). Chronically poor areas have experienced disinvestment and exploitation for several years. Between 1990 and 2015, one-third of the young adult (ages 25–34) population in these areas had left. Only about one-third of working-age adults are working in chronically poor areas. Geographic and social isolation is pervasive (122). Ulrich-Schad & Duncan surveyed residents in a sample of these types of rural communities between 2007 and 2013 and found that significant differences exist between these three categorizations in community concerns and in economic, social, and political indicators.

More recently, several efforts have expanded the measurement of rural by building on the concept of a continuum and adding an asset and strength-based component to the understanding of rural areas. The index of relative rurality creates a continuum of classification from very low rurality to very high rurality (128), and the continuous geographic isolation scale identifies the extent of isolation of various locations (33). The rural comprehensive wealth framework broadens the indicators of wealth to include the following types of capital (as assets): financial, physical, human, intellectual, social, political, cultural, and natural. Each contributes to population well-being (61). Strength-based approaches highlight positive characteristics, including self-determination and autonomy, local ways of knowing, comprehensive wellness (rather than biomedical illness), root cause analyses of structural determinants of health, and hope (41).

## RURAL HEALTH DISPARITIES

Rural health disparities have attracted increased national attention, and research shows that disparities in rural health outcomes exist (45, 84). When considering the identified disparities, however, it is important to note the challenge of small sample sizes in this research. For instance, many of the national surveillance tools can include only data at the county level. Counties are often not a sufficient proxy for “rural” (e.g., when a county includes rural and nonrural communities). When case numbers are low, county-level numbers are often suppressed, owing to privacy considerations (68). Certain subgroups within rural areas may be underrepresented in surveillance systems as a result of limitations in data collection methods (e.g., timing of data collection, use of landline phones) (4, 69, 101). Finally, survey items used in research may not be culturally relevant or reflect the needs and priority concerns of rural populations (4, 101).

Despite these challenges, the literature documents a number of clear rural health disparities. For example, age-adjusted rates for nonmetropolitan areas are higher for all-cause mortality, labeled a “rural mortality penalty” (24). The five leading causes of death in the United States—heart disease, cancer, unintentional injuries, stroke, chronic lower respiratory disease—are higher for nonmetropolitan (rural) areas than for metropolitan areas (often used to connote urban) (45, 84, 117). Furthermore, greater percentages of these deaths in nonmetropolitan areas are considered excess deaths than in metropolitan areas (45, 84). In addition, all-cause mortality has increased in rural areas over time (1999–2001 versus 2013–2015) while decreasing in urban areas (117), and the disparity in death rates between nonmetropolitan and metropolitan areas for some conditions, such as for heart disease and chronic lower respiratory disease, increased between 2010 and 2017 (84).

A variety of determinants, such as health risk behaviors and health care access, as well as socioeconomic and environmental determinants, influence these differences in mortality among urban and rural areas (78, 84). Rural residents were less likely than were their urban counterparts to be current nonsmokers, to be meeting current aerobic physical activity recommendations, and to be at normal body weight and were less likely to have insurance and screening for breast, cervical, or colon cancer (47, 56, 78). Environmental risk factors also differentially affect rural versus urban residents. Although rural areas tend to have better air quality, they have worse water quality (119); food deserts are more prevalent in rural areas (75); and exposure to vapor-gas, dust, and fumes was almost three times higher in rural workers (32). Socioeconomic factors include changing job markets; for example, farming and mining jobs have decreased significantly between the 1970s and 2000s (9, 122). And while many rural adults used to work in one full-time job, many are now having to work in multiple part-time jobs—often in economically vulnerable industries—to be able to sustain the same level of financial resources as they had in the past (56, 122). Compared with urban families, rural families have lower median incomes, higher rates of food insecurity and of homelessness, and less access to transportation or other resources needed to access services (56). Over 60% of rural counties experience persistent poverty, four times more than nonrural counties (47). Public spending on social services, such as education and parks, in rural areas is significantly lower than that in urban areas (71).

Health disparities extend across the lifespan and are also evident among children (99). In 2017, annual death rates for rural children (birth to 19 years of age) were 25% higher than those for urban children. With respect to cause-specific mortality, rural children (1–19 years of age) had higher rates of suicide and unintentional injuries than did urban children, whereas urban children had higher rates of assault deaths (99). Rural children were more likely to be overweight than their urban peers. Adolescents living in rural areas were more likely to report that they had had sex in the past 3 months; had used tobacco, alcohol (including binge drinking and driving under the influence), and cocaine; and had given birth as compared with their urban counterparts (100).

### The Intersection of Race and Place

Racial/ethnic disparities are also evident within rural areas. Place and race intersect. Recent descriptions of deaths of despair have highlighted a reversal of mortality declines for White Americans aged 25–54 years who live in rural areas. This change is attributed to increasing rates of suicides, liver diseases, and poisonings (including overdoses from opioids) (16, 117). Though this reversal is critical to address, it is important to note that overall mortality rates of Black Americans who live in rural areas remain higher than those of their White counterparts (30, 117), and the reversal of mortality declines has also begun for other racial groups (131). Socioeconomic conditions of Black, Hispanic, and Native populations living in rural areas are also worse than those of their non-Hispanic White counterparts (59); a significantly greater percentage of Black, Hispanic, and Native people report having less than a high school–level education and annual household incomes less than $25,000. Black and Native populations are also less likely to be employed (59).

Several health-related morbidity outcomes are also worse for Black, Hispanic, and Native populations living in rural areas compared with their non-Hispanic White counterparts (59, 105). A significantly greater percentage report their health status as fair or poor, that they have obesity, and that they have not been able to see a doctor in the past year owing to cost; significantly fewer people state that they have at least one personal health care provider and meet colorectal cancer screening recommendations (59). Black and Native rural residents are also more likely to state that they had frequent physical and mental distress in the past month and to report having two or more chronic conditions as compared with their White non-Hispanic counterparts (59). White non-Hispanic rural residents are more likely to report current smoking and binge drinking than were their Black and Hispanic counterparts (59). More recent scholarship highlights the necessity for considering intersectionalities (e.g., rural, black, female) to inform a deeper understanding of rural well-being (46). Indeed, rural residents of color had worse health outcomes than did urban residents of color (105).

### The COVID-19 Pandemic in Rural Areas

Given the timing of this manuscript’s publication, we would be remiss to ignore the coronavirus 2019 (COVID-19) pandemic and its specific impact on rural spaces. The COVID-19 pandemic has affected all areas of the United States. Much of the narrative of the impact of COVID-19 has centered on urban areas, with less attention given initially to the influence of rurality (85). Rural areas experienced the pandemic differently than did urban areas, and rurality may be a critical axis of explanation for the pandemic’s impact (85). While rural areas had several characteristics that may have inhibited the spread of the virus, such as a lower population density, they had other attributes that resulted in higher burden. These included higher levels of poverty; fewer employment opportunities and more work in jobs that were affected by COVID-19-related closure; and an older population: About two-thirds of small metropolitan areas were classified as older age counties compared with only 7% of large metropolitan counties (83, 85). This population also experiences more chronic diseases, which are a significant risk factor for severe COVID-19 consequences (56). The COVID-19 pandemic disproportionately affected Black, Latinx, and Native populations across the United States (97, 120), and rural areas were no exception (132). In addition, access to health care is limited in rural areas, which has meant limited access to testing (83, 85) and to intensive care unit (ICU) care, as “only 1% of the nation’s ICU beds are located in rural areas” (83, p. 2). In response, rural health clinics began offering telemedicine services; up to 97% of surveyed rural health clinics and critical access hospitals reported offering these services (56). However, broadband issues persist in rural areas, further limiting access to health services; up to one-third of people living in the rural United States do not have adequate Internet to allow telemedicine visits (56). Physicians in rural areas are also older and therefore themselves more at risk for COVID-19 infection (83).

Without consideration of these aspects of rurality that determine the pandemic’s impact on the population, mitigation measures were and will continue to be less effective (85). The Kaiser Family Foundation is tracking COVID-19 vaccine intentions across the United States and has found that more rural residents than urban residents have taken a wait-and-see attitude toward vaccination or are not intending to be vaccinated (67). This finding has been confirmed by lower COVID-19 vaccination rates in many rural as compared to urban counties (87). Although the language of vaccine hesitancy or confidence focuses on individual attitudes, these attitudes may be influenced by histories, context, and political ideology (1, 23, 95).

The COVID-19 pandemic further highlighted the symbiosis of rural and urban areas. As noted by Monnat (83),

The impacts of the coronavirus epidemic on rural communities will also have major implications for urban populations. Rural America supplies disproportionate shares of the nation’s food, energy, military personnel, and natural amenity recreation. These are resources that urban America depends upon. Rural, urban, or somewhere in between—we are all in this together.

(p. 4)

COVID-19 has also partially flipped the narrative of the rural–urban divide and confirmed the complexity of definitions of space. Previous to the pandemic, many urban dwellers viewed rural areas through a deficit lens of negativity and deficiency (74), but the threat of long periods of lockdown and crowded cities resulted in some exodus of upper- and upper-middle-class urban residents to rural areas. Rural locations became places of wanted escape from the pandemic, leading to “disaster gentrification” of rural spaces (77). In the context of COVID-19, Rich (104) and Malatzky et al. (77) suggest that urban dwellers made individual rights choices with little regard for the collective impact on rural communities. These choices by urban dwellers could be perceived as disrespecting and devaluing rural residents’ ability to maintain their lives.

The disparities highlighted in this and the previous section have resulted from a long history of a lack of attention to rural areas and a focus on a deficit narrative. A deficit lens describes a group of people or a place only in terms of deficiencies, failure, problems, limitations, and negativity (31, 41). This perspective results in personal and social harms to the group in question and has the potential to limit scientific progress (31). Both the lack of attention to rural areas and a deficit lens may influence health care provider shortages, thus increasing health disparities (26). Disparities also result from a focus on proximal (e.g., knowledge, behavior) instead of distal (e.g., social and structural determinants) causes. Reimagining rural requires shifting to a strength-based paradigm (as described above) and an emphasis on structural determinants, as well as a local voice.

## SHIFTING PARADIGMS: A HEALTH EQUITY APPROACH TO RURAL HEALTH AND WELL-BEING

**Figure 1:**
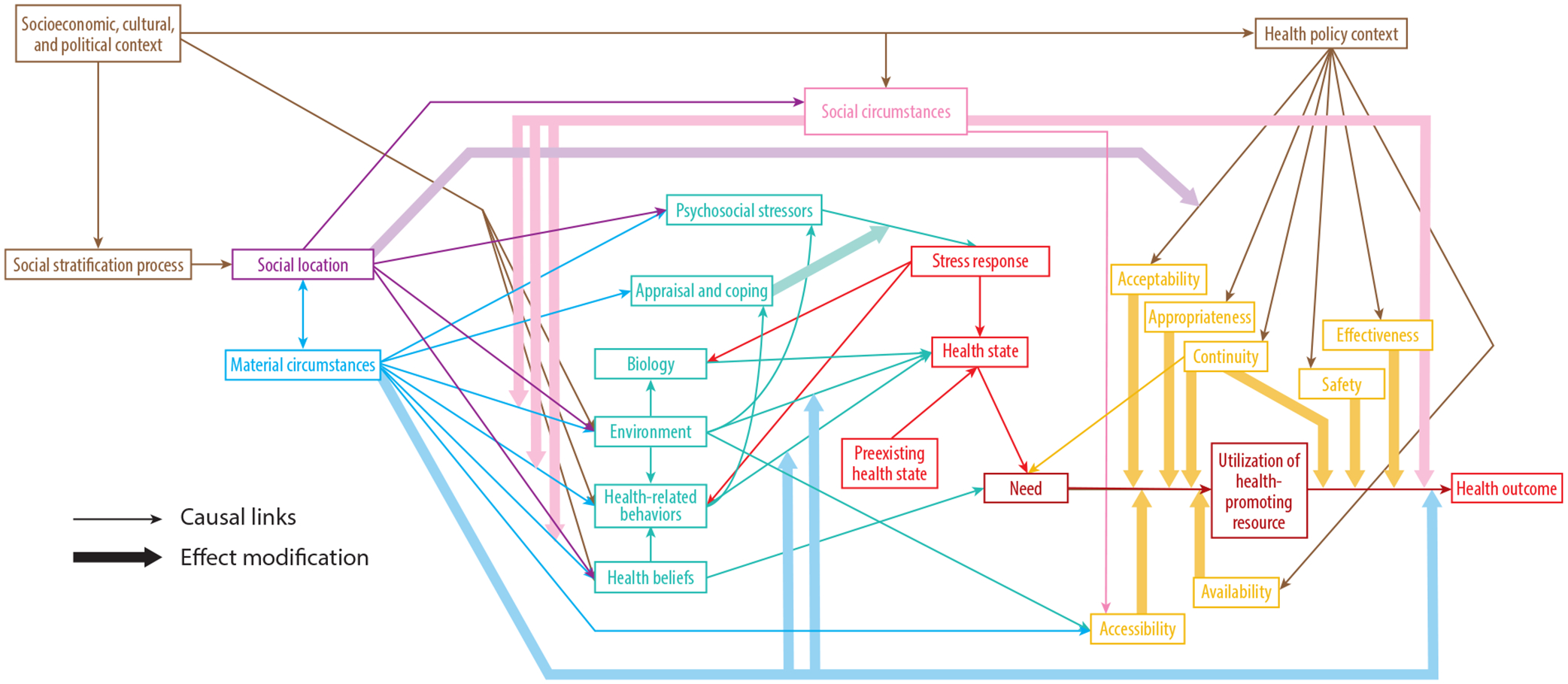
Health equity measurement framework (HEMF). The HEMF illustrates critical pathways linking socioeconomic, cultural and political contexts (SECPC) to (in)equitable health outcomes. By centering social stratification processes and impacting health policy, SECPC initiates a cascade of intersecting effects influencing our health. Figure first published in Dover & Belon (34) (CC BY 4.0); correction published in Dover & Belon (35).

Given the documented health disparities of rural areas, a paradigm shift is needed that centers the complexity and strength of rural places to advance health equity. Health equity frameworks have been identified as addressing inequities by shifting attention from deficits to what society can do to maximize opportunity for all (14). Dover & Belon’s (34, 35) health equity measurement framework (HEMF) (Figure 1) expands on previous frameworks and may be valuable in shifting the paradigm of rural health.

The HEMF postulates that the socioeconomic, cultural, and political context (SECPC) is a “powerful determinant in the formation and reproduction of social structure and a driving force in policy development and implementation” and therefore “has an impact on social distribution of health and people’s opportunities to be healthy” (35, p. 3). Key concepts in the HEMF framework include social stratification processes, which result from the distribution of power, resources, and prestige; social location, which refers to one’s relational position in the hierarchy of society; material circumstances, which influence social circumstances such as social cohesion and social capital, psychological stressors, environments, health-related behaviors, and health beliefs; and the health policy context, which creates an enabling environment for health equity through enhancing health system quality. Other variables in the HEMF include need, response to stress, biology, and utilization of health-promoting resources.

The relationship between these variables is complex; the SECPC drives the social stratification processes and the health policy context. Social stratification processes then influence social location. Social location influences material circumstances, which affect environments in which people are born, live, work, play, and die; social circumstances; stress and coping mechanisms; and health-related behaviors and health beliefs. Social location also influences the implementation of the health policy contexts through the acceptability, appropriateness, continuity, effectiveness, and safety of policy options. In the HEMF, social circumstances play a moderating effect on most other variables. Though seemingly complex, the HEMF focuses attention on structural determinants, such as social, economic, and political decisions, and the way they seep into bodies and systems (35). By identifying and elucidating how SECPC impacts all elements of the framework and thus health, the HEMF is a valuable tool by which to understand the cascade of power and privilege to influence health (in)equity and the “broader institutional and structural contexts that constrain choice and…reproduce oppression” (105). Reimagining rural health might benefit from this paradigm shift for research and practice.

The structural factors identified in the HEMF are evident in the historical and current limited investment in rural places (47, 106). In the United States, rural areas have historically had less access to public health services than have urban areas. The first rural health department (funded mostly by private foundations) was established in 1912, 115 years after the first city board of health (Philadelphia was the first city to establish a board of health in 1797). Rural public health departments shifted from disease prevention to the provision of basic health care services around 1930, and, to date, these services remain the core services provided, owing to an otherwise lack of access to health care for rural residents (47, 81). A more comprehensive view of health-promoting systems in rural areas still appears to be far away (81); the current system symbolizes the lack of perceived economic and social value of rural places (64).

Whereas the percent of the US population that lives in rural areas was 19% (123), less than 20% of physicians, nurse practitioners, and physician assistants worked in rural areas (2). As of March 2021, 62% of primary medical health profession shortage areas (HPSA), 63% of dental HPSA, and 58% of mental HPSA were rural (55). And “the 1,500 smallest and most rural LHDs (local health departments) in the country have fewer staff than the largest 25 LHDs combined” (47, p. 175). In the area of public health research and intervention design, rural areas have often been left to adapt interventions designed for urban settings, with little or no consideration of the appropriateness of those programs for a rural setting (17, 113, 127).

Malatzky & Bourke (76) describe how the SECPC impacts health in rural settings. Though their analysis focuses on Australia, the themes echo in the status of rurality in the United States (105). Applying a Foucauldian analysis, the authors deconstruct the power and discourse that has resulted in the current status of how rural is viewed, noting that while the concepts of rural and urban are socially and sociopolitically constructed, they have framed scientific inquiry. Power normalizes a particular discourse around a phenomenon and imposes homogeneity as an instrument of that power and discourse (76). As noted previously, the deficit discourse pervades our social understanding of rural, which has been homogenized to ignore nuance and heterogeneity, despite data suggesting that the latter is the case. “Rural health is presented for what it lacks compared to urban areas rather than what it achieves in its own context” (76, p. 158). As an example, Malatzky & Bourke (76) point to the narrative around an element of the social stratification HPSA and how the narrative focuses on a limited supply of human resources for health in rural areas rather than on a maldistribution of resources overall. Ultimately, the dominant narrative of rural spaces reflects the SECPC elements of the HEMF framework, underlying how social, economic, and political discussions and decisions drive the resource allocation and consequent health disparities in rural areas and therefore impacts “people’s opportunities to be healthy” (35, p. 3; 64; 104).

The centering of SECPC and the deconstruction of power and narrative have the potential to result in interventions that advance equity by considering the context of rural areas, giving voice to the residents of rural areas, and acknowledging the complexity of adapting and implementing interventions into rural settings.

## IMPLEMENTATION SCIENCE, HEALTH EQUITY, AND RURAL HEALTH AND WELL-BEING

Evidence-based public health (13) posits that all interventions must be guided by the intersection of “scientific evidence and values, resources, and context” (13, p. 177). Yet, even when interventions have been proven effective, their dissemination is limited. This reality has resulted in an emphasis on implementation science (IS), which seeks to understand the implementation processes required to diffuse effective transformational interventions. Implementation science is “the scientific study of methods to promote the systematic uptake of research findings and other evidence-based practices into routine (community or health service) practice” (36, p. 1). Though IS, as a label, became popularized in the 1990s, its origins can be traced back much further to Lewin’s approach of action research and Roger’s theory of diffusion of innovations (40). The basis of IS in diffusion of innovations is particularly relevant to our discussion as it arose from the work of rural sociologist Everett Rogers (107) and the diffusion of agricultural innovations in rural social systems. Key features of successful diffusion in agriculture, which remain critical to IS, include the relative advantage of the innovative practice over the current one and the ability to try out the innovative practice prior to rollout, among others (73, 109). The significant gap between knowledge of effective interventions and their scale-up and sustainment to promote population health is the impetus for the work of Lewin, Rogers, and others and the current emphasis of IS.

IS begins with an evidence-based intervention (EBI) and focuses on the rollout of the intervention within a context, including the intervention’s acceptability, adoption, appropriateness, feasibility, fidelity, implementation cost, coverage, and sustainability (96). To respond to the contextual aspects (rural and otherwise) of EBI rollout, IS has emphasized the importance of adapting interventions to those contexts. A recent scoping review of frameworks for adapting public health EBIs found 13 adaptation frameworks that suggested 11 steps for program adaptation (38). Ongoing and iterative adaptation is critical (21), particularly related to the nuanced nature of rural areas; what works in one rural area may not work in another.

While IS is crucial for a more evidence-based approach to public health, there are several challenges inherent in applying IS to rural areas. Very few EBIs have specifically been developed in and for rural areas or in response to the diversity of rural areas (115; https://www.thecommunityguide.org/). Compounding this dearth of interventions, a systematic review of the implementation of EBIs globally found only three studies that specifically *adapted* EBIs to rural contexts in the United States (37). In addition, as noted above, rural health departments have a lower budget and, consequently, fewer programmatic resources and staff. Perhaps owing to these two factors, rural health departments were found to be less likely to implement evidence-based decision making (47). In line with the power and discourse analysis described above, rural areas are expected to implement EBIs developed and evaluated in urban spaces and to do so with far fewer resources. Challenges in implementing these EBIs in rural settings have included cultural misfit, practical limitations, lack of commitment and perceived lack of applicability on the part of rural practitioners and partners, insufficient capacity, and unfavorable policy conditions (115). In addition, implementation of EBIs in LHDs has been found to be contingent on specific administrative evidence-based practices (A-EBPs) related to workforce development leadership, organizational climate and culture, relationships and partnerships, and financial processes (15). Further confirming the power and discourse analysis described above, smaller health departments in rural areas were found to have lower performance on these A-EBPs than did larger urban health departments, thus limiting implementation of EBIs in rural settings (15).

Although an understanding of the SECPC is a key principle in IS (96), “theories, models and frameworks used in implementation science” (90) had not directly taken on health equity. More recently, IS has embraced the call to advance health equity. Several authors provide guidance for centering health equity in IS (8, 14, 22, 39, 80, 111, 112). Some of these authors recognize that achieving health equity goals will require IS researchers to sit with discomfort in applying methods that are less reductionist and embrace the messiness and complexity of health equity models that center the historical, economic, political, and social forces that impact well-being (8, 14). Yet, none of these IS health equity guidance documents or frameworks specifically call out rural disparities or apply an intersectional lens.

## COMMUNITY-ENGAGED RESEARCH AND PRACTICE IN RURAL SPACES: A TOOL IN THE PARADIGM SHIFT

Adaptation of EBIs and equity-centered IS is not possible without authentic and meaningful community participation (8, 14, 80). Community-engaged approaches involve community members in various ways in the definition of the issue and the planning, implementation, and evaluation of any program or research activity. Community-engaged approaches fall on a continuum from more tokenistic to fully participatory (66, 79, 94). Health equity–based engagement favors shared leadership and community-driven and -led processes. The values of community-engaged praxis (116) and the principles of community-based participatory research (CBPR) (58) include health equity and social justice as central premises. Participatory praxis also has the potential to flip power dynamics, thereby allowing groups who have experienced marginalization as a result of oppressive systems, structures, and SECPC to gain greater control and self-determination over their lives and environments (116). Community-engaged processes also enhance the rigor, reach, and relevance of interventions (5, 88).

Yet, equitable community participation is not always present in rural health research, even those that take a community-engaged approach. A scoping review of community participation in rural health (65) found only six articles globally that engaged participants at the level of shared leadership; three of these were based in rural areas of the United States: in California, Louisiana, Tennessee, and Virginia (12, 57, 63). Results indicated that—in all six projects—despite high levels of coleadership, decision-making power remained with the health professionals and academic institution, as these parties held the financial resources. One pertinent takeaway was the suggestion that limited resources within rural areas may provide incentives to work collaboratively. Implementing community-engaged research in rural areas with increasing diversity must also consider balancing attention to the different experiences of different health disparities populations (rural-only disparities versus rural intersected with race, ethnicity, or gender disparities) (93) and reflecting on the opportunities for meaningful engagement with newly arrived community members in new destination communities (72, 93). The importance of an “equity-based participatory implementation science approach” was highlighted as an opportunity to promote rural cancer control (129, p. 346).

## FUTURE CONSIDERATIONS FOR RURAL RESEARCH AND PRACTICE

Throughout this article, we have deconstructed the definitions and narratives of rural communities and have suggested a necessary paradigm shift to uplift the complexity of health and well-being in the rural United States. With that in mind, we offer the following suggestions to enhance rural research and practice.

### Change the Paradigm to Recognize the Strengths and Challenges of Rural Areas

We argue that the dominant approach to understanding rural has focused on a monolithic (and mostly negative) imaginary of rural spaces that is minimalist. This narrative results from macro social, economic, and political processes (6) that influence much of the research and practice around rural health and well-being, as well as social and health policy directed at rural areas. Furthermore, this narrative minimizes the complexity and diversity of rural spaces and erases the vitality of rural residents. Reimagining rural requires a paradigm shift that centers health equity and local voice and flips the narrative to uplift assets and strengths of rural communities. Implementing this vision will first require an acknowledgment of our own assumptions and biases about rural areas. Researchers and practitioners engaging with rural communities may consider employing cultural humility (52) and recognize their power and privilege and how those impact and influence their choice of approaches, tools, and methods (102).

### Address the Challenges of Data Collection in Rural Areas

In addition to uplifting assets and strengths, data collection and analysis in rural areas could be enhanced in several ways to ensure accurate representation of rural communities. Mixed-methods research using a CBPR approach may provide opportunities to collect valid robust data (4, 69, 101). Others have suggested ways to make better use of existing data as well as new data collection methods (4, 19, 92).

### Recognize and Address Limits of IS in Rural Research

Guidance on interventions that work in rural areas does exist but is limited (e.g., 11, 18; https://www.ruralhealthinfo.org/project-examples/evidence-levels/evidence-based). The Centers for Disease Control and Prevention funds Prevention Research Centers (PRCs) across the United States, which develop, test, and/or evaluate public health interventions. Several PRCs focus on rural areas and adaptations of EBIs to these settings (e.g., 3, 7, 20, 29, 53, 70, 118, 130).

While IS is an obvious approach for increasing the availability and reach of EBIs (often tested in urban contexts) for rural areas, the differences in culture, geography, and traditions between urban and rural areas will require a much more intentional focus on developing unique interventions for this context. This effort could focus on acknowledging the range of rural (rather than one view of rural), rather than simply modifying urban interventions, and on transferring lessons learned from rural areas globally to the United States. IS methods and tools (https://prevention.nih.gov/research-priorities/dissemination-implementation; https://www.fic.nih.gov/About/center-global-health-studies/neuroscience-implementation-toolkit/Pages/resources.aspx; http://adphealth.org/irtoolkit/) can enhance our understanding of the facilitators and challenges of adopting/adapting EBIs from one rural setting to another, rather than from an urban setting.

### Extend IS Health Equity Frameworks to Include Rural Places and Intersectionality

As noted above, while critical readings on IS and health equity rightly emphasize race, ethnicity, sexual orientation, ability, and socioeconomic status, they do not include place. Given our understanding of the importance of place in constructing inequities, the clear evidence of specific inequities in rural health outcomes, and the diversity of rural places, we suggest that rurality be considered in IS health equity frameworks.

Furthermore, acknowledging the historical and increasing diversity of rural areas, an intersectional health equity focus is imperative for IS research and practice. Intersectionality, originally coined by legal scholar K. Crenshaw (25), suggests that individuals carry multiple identities and that each or any could confer advantage or disadvantage. “Intersectionality…emphasizes that sociodemographic attributes interact with social, political, regulatory, and contextual factors to shape privilege, power, and the lived experiences among individuals at certain sociodemographic intersections that may increase the risk for health inequities” (98, p. 116). We argue that rurality is an additional axis of intersectionality.

### Bring Rural Voices into the Design and Implementation of Research and Programming Through a Community-Engaged Approach

As noted previously, the voice of rural residents has not always been included in defining what is rural or in designing solutions to rural health problems. Community participation is particularly important in the design of and/or adaptation of EBIs and equity-centered interventions (https://engageforequity.org/). Combining many of the recommendations above, a recent IS framework suggests a transcreation approach as a tool to achieve health equity goals (88). The transcreation framework for community-engaged behavioral interventions to reduce health disparities begins with strengths and resources found in communities and emphasizes the knowledge already within communities as critical to the adaptation of EBIs. However, the framework acknowledges that “when researchers address social determinants and health disparities with the full engagement of community partners experiencing the disparity, the intervention produced is not an adapted EBI, but a new intervention because of the extensive adaptations required to fit the community context in the presence of these disparities” (88, p. 11).

### Commit to Health Equity by Reflecting on Limitations of Standard Methods and Approaches

We have argued for the critical importance of addressing structural determinants of health to advance health equity in rural areas. However, impacting the social, economic, and political contexts that influence rural health will require researchers and practitioners to extend beyond the linear thinking that makes research “cleaner” to embrace frameworks and theories that model the complexity of the places where people are born, live, learn, work, play, worship, and age. These might include systems science (44), syndemics (114), critical race theory (42, 43), life course approach (62), intersectionality (50), feminism (28, 108), and chaos and complexity theory (103, 121). A recent call to the IS field provides three “key recommendations to actively consider structural racism in research questions, frameworks, methods, measures, and strategies for health equity”: embedding a consideration of structural racism in all aspects of the research, utilizing multilevel approaches, and engaging in transdisciplinary and intersectoral partnerships (111).

## CONCLUSION

Rural health should be recognized for its contextual nuances, its assets, and its glaring health disparities. To do so, a new paradigm is needed to allow for a focus on strengths and health equity. IS can help with the programming of this paradigm, but limitations of IS must be considered and addressed. Focusing on rural in its own right will lead to intervention innovations and reinvention that have implications beyond rural areas. As a result, addressing rural health and its complexity will contribute to the reduction of health inequities in the United States. We offer up these suggestions to inspire renewed interest in partnering with rural communities to promote health equity.

## SUMMARY POINTS

1. Dominant representations of rural places suggest a singular deficit-based place, but rural places are diverse and nuanced. Understanding the complexity of rural places is critical because that knowledge drives social and health policy and services.
2. Death rates from the five leading causes of death in the United States are higher for rural than urban areas. Rural health disparities affect children as well as adults. Racial/ethnic disparities are also evident within rural areas. Place and race intersect.
3. Rural health disparities have resulted from a long history of a lack of attention to rural areas and a focus on a deficit narrative. Reimagining rural requires a shift to a strength-based paradigm and an emphasis on structural determinants and local voice.
4. Health equity frameworks shift attention from deficits to what society can do to maximize opportunity for all. The health equity measurement framework highlights the importance of considering the social, economic, and political context when assessing health outcomes and developing interventions.
5. Implementation science (IS) aims to promote the uptake of evidence-based interventions (EBIs) in practice. But very few EBIs have been developed specifically in and for rural areas or respond to the diversity of rural areas. Furthermore, few EBIs have been adapted to rural areas.
6. IS has recently embraced the call to advance health equity and provide guidance on centering equity in IS frameworks and methods. Yet, none of these IS health equity guidance documents or frameworks specifically call out rural disparities or apply an intersectional lens.
7. Adaptation of EBIs and equity-centered IS is not possible without authentic and meaningful community participation. Bringing rural voices into the design and implementation of research and programming through a community-engaged approach is critical to advance rural well-being.
8. Focusing on rural in its own right—and committing to health equity—will lead to intervention innovations and reinvention that have implications beyond rural areas. We hope to inspire renewed interest in partnering with rural communities to promote well-being.
